# ﻿New species of *Vamsapriya* (Vamsapriyaceae, Xylariales) and *Neolinocarpon* (Linocarpaceae, Chaetosphaeriales) from Yunnan, China

**DOI:** 10.3897/mycokeys.120.156619

**Published:** 2025-07-29

**Authors:** Xingyu Luo, Changtao Lu, Kamran Habib, Yulin Ren, Yilan Jin, Wenmei Li, Pingzhu Lu, Yingqian Kang, Xiangchun Shen, Hind A. Al-Shwaiman, Abdallah M. Elgorban, Nalin N. Wijayawardene, Yang Chen, Qirui Li

**Affiliations:** 1 State Key Laboratory of Functions and Applications of Medicinal Plants, Guizhou Medical University, University Town, Guian New District, Guiyang, Guizhou province, 561113, China; 2 The High Efﬁcacy Application of Natural Medicinal Resources Engineering Center of Guizhou Province (The Key Laboratory of Optimal Utilization of Natural Medicine Resources), School of Pharmaceutical Sciences, Guizhou Medical University, University Town, Guian New District, Guiyang, Guizhou province, 561113, China; 3 Anshun University Agricultural College, Anshun, 561000, China; 4 Chongqing Three Gorges Medical College, Wanzhou Wanzhou District, Chongqing, 404100, China; 5 Key Laboratory of Environmental Pollution Monitoring and Disease Control, Ministry of Education, School of Basic Medical Sciences, Guizhou Medical University, Gui’an New District, Guiyang, 561113, China; 6 Add Department of Botany and Microbiology, College of Science, King Saud University, P.O. 2455, Riyadh 11451, Saudi Arabia; 7 Center of Excellence in Biotechnology Research (CEBR), King Saud University, Riyadh, Saudi Arabia; 8 Center for Yunnan Plateau Biological Resources Protection and Utilization, College of Biology and Food Engineering, Qujing Normal University, Qujing, Yunnan 655011, China

**Keywords:** Ascomycetes, bambusicolous fungi, fungal systematics, new species

## Abstract

During a survey of ascomycetes in southwestern China, three new bambusicolous fungi were collected and identified as members of the genera *Neolinocarpon* and *Vamsapriya*. The newly identified species are designated *Neolinocarponbambusicola***sp. nov.**, *Vamsapriyatongluobaensis***sp. nov.**, and *V.zhaotongensis***sp. nov.**, delineated through an analysis of both morpho-anatomical characteristics and multi-locus phylogenetic analyses based on ITS, LSU, *rpb2*, and *tub2* sequence data. The distinction of these species from their known counterparts was verified through comparisons of morphological characters and phylogenetic analyses. Comprehensive morphological descriptions, illustrations, and a phylogenetic tree showing the placement of the new taxa are provided.

## ﻿Introduction

The genus *Vamsapriya* was introduced by Gawas and Bhat (2005) to accommodate synnematous hyphomycetes colonizing bamboo in India, with *V.indica* designated as the type species. Dai et al. (2017) described the first sexual morphs of *Vamsapriya* and placed the genus within Xylariaceae, as accepted by Hyde et al. (2020). Later, multi-locus phylogenetic analyses by Sun et al. (2021) revealed that *Vamsapriya*, along with its closely related genera *Diabolocovidia* and *Didymobotryum*, formed a distinct monophyletic clade within Xylariales, clearly separated from the Xylariaceae. This finding led to the establishment of the new family Vamsapriyaceae, with *Vamsapriya* designated as the type genus. The asexual morph of *Vamsapriya* is characterized by colonies on natural substratum that are black, mycelium immersed, septate, branched, conidiophores macronematous, synnematous, erect, dark brown, cylindrical, synnemata with cylindrical to clavate apical fertile part composed of compactly arranged conidiophores; conidiogenous cells monotretic, integrated, terminal, brown, cylindrical to clavate; conidia catenate, acrogenous, cylindrical, broadly fusiform, or obclavate, and brown to dark-brown conidia (Sun et al. 2021). The sexual morph of the genus is characterized by black perithecial, immersed ascomata, sub-globose, ostiolate, surrounded by dark brown peridium, 8-spored short pedicellate asci, each with a J+ apical ring and fusiform to broadly fusiform, apiosporous ascospores, hyaline, pointed at both ends, with or without a mucilaginous sheath (Sun et al. 2021). Fourteen species of *Vamsapriya* have been documented worldwide, but only eight species of the genus are known to possess a sexual morph (Dai et al. 2017; Sun et al. 2021; Samarakoon et al. 2022; Dissanayake et al. 2024; Liu et al. 2025). Species of the genus are predominantly found in tropical and subtropical regions, with records from China, Cuba, India, and Thailand, with the majority of species reported from Thailand and China. Ecologically, *Vamsapriya* species are primarily saprobes on decaying bamboo culms (Sun et al. 2021; Samarakoon et al. 2022; Dissanayake et al. 2024; Liu et al. 2025).

The genus *Neolinocarpon* was introduced by Hyde (1992a) to accommodate many *Linocarpon*-like species, with *N.globosicarpum* as the type species. The genus is characterized by a clypeus with a dense, blackened, shiny central papilla; deeply immersed, oval to globose ascomata; 8-spored unitunicate asci, each with a reflective apical ring; and filiform, fasciculate ascospores containing refringent bands, with or without appendages (Hyde 1992a, b; Hyde et al. 1998; Vitória et al. 2013; Konta et al. 2017). *Neolinocarpon* is morphologically similar to *Linocarpon*, particularly in the apical structure of the ascus and ascospore morphology, but differs in having deeply immersed ascomata that form below a slightly raised or flattened clypeus, with a refractive globose body beneath the ascus apical ring (Hyde 1992b). Nearly all species of *Neolinocarpon* are found on hosts in the Arecaceae, except for *N.penniseti* and *N.phayaoense*, which occur on hosts from Poaceae and Euphorbiaceae, respectively (Hyde 1992b; Hyde et al. 1998; Hyde and Alias 1999; Bhilabutra et al. 2006; Vitória et al. 2013; Senwanna et al. 2018). As of March 10, 2025, Index Fungorum lists 13 species in this genus (www.indexfungorum.org).

During a mycological investigation of ascomycetes associated with decaying bamboo in Yunnan Province, Southwestern China, we found three novel saprobic bambusicolous fungi belonging to the genera *Vamsapriya* and *Neolinocarpon*, each with distinct morpho-anatomy that does not fit with any of the previously described species within their respective genera. Saprobic fungi play an important role in the natural breakdown of organic materials and often harbor biotechnologically relevant enzymes and secondary metabolites with potential applications in agriculture, medicine, and environmental management (Corbu et al. 2023). This study provides the basis for future investigations into these fungi, which may lead to valuable applied research and potential biotechnological innovations.

## ﻿Materials and methods

### ﻿Sample collection and morphological study

The specimens were collected during surveys conducted in Yunnan Province in China from August to November in 2024. All related habitat information was recorded. Photographs of the collected materials were taken using a Canon G15 camera (Canon Corporation, Tokyo, Japan). Materials were placed in paper bags and taken to the lab for morphological characterization and isolation. The specimens were placed in a ventilated area at room temperature for drying. All specimens were deposited at the Herbarium of Guizhou Medical University (**GMB**) and the Herbarium of Cryptogams, Herbarium of Kunming Institute of Botany, Chinese Academy of Sciences (**KUN HKAS**). Living cultures were deposited at the Guizhou Medical University Culture Collection (**GMBC**). All scientific names of fungi follow the entries in MycoBank and Index Fungorum; hence, no authorities and year of publication are given in the text.

### ﻿Morphological characterization and isolation

Macroscopic characteristics were examined under an Olympus SZ61 stereomicroscope (Japan) and photographed with a Canon 700D digital camera (Canon Inc., Tokyo, Japan). The morphological features were studied as described by Li et al. (2024a). The samples were mounted in water for microscopic examination; Melzer’s reagent was added when necessary. The Tarosoft Image Framework (v.0.9.7) program and Adobe Photoshop CS6 software (Adobe Systems, USA) were used for measuring and processing images. Axenic cultures were obtained from single spores or tissues as described by Senanayake et al. (2015). Pure cultures on potato dextrose agar (PDA) were isolated using single-ascospore isolation techniques (Liu et al. 2025). The cultures were incubated at 25–30 °C for 4–6 weeks, with weekly observations of growth and development (Senanayake et al. 2020).

### ﻿DNA extraction, PCR amplification, and sequencing

Mycelium was scraped from pure culture plates using a sterilized scalpel and used for DNA extraction following the manufacturer’s instructions for the BIOMIGA fungus genomic DNA extraction kit. The DNA samples were kept at –20 °C. Internal transcribed spacer region (ITS), large subunit (LSU), ribosomal small subunit (SSU), the second largest subunit of RNA polymerase II (*rpb2*), and β-tubulin (*tub2*) were amplified by PCR with primers ITS1/ITS4 (White et al. 1990; Gardes and Bruns 1993), LR0R/LR5 (Vilgalys and Hester 1990), NS1/NS4 (White et al. 1990), fRPB2-5F/fRPB2-7cR (Liu et al. 1999; Bischoff et al. 2006), and Bt2a/Bt2b (Glass and Donaldson 1995), respectively. The components of a 25 μL PCR mixture were 9.5 μL of double-distilled water, 12.5 μL of PCR Master Mix, 1 μL of each primer, and 1 μL of template DNA. The PCR amplification was conducted as reported by Samarakoon et al. (2022). PCR products were checked through 1.5% agarose gel electrophoresis stained with Golden View and were sent to Sangon Co., China, for sequencing.

### ﻿Sequence alignments and phylogenetic analyses

All the obtained sequences were compared with known sequences in GenBank using the BLAST algorithm for precise identification (https://www.ncbi.nlm.nih.gov) (Altschul et al. 1990). The reference sequences retrieved from open databases originated from recently published data (Liu et al. 2025) and the BLASTn results of close matches. Sequences were aligned using the MAFFT v.7.110 online program (Katoh et al. 2019) with default settings. The alignments were adjusted manually using BioEdit v.7.0.5.3 (Hall 1999) where necessary. Maximum likelihood (ML) analyses were implemented in RAxML v.8.2.12 using the GTRGAMMA substitution model with 1,000 bootstrap replicates (Stamatakis 2014). Phylogenetic analyses were also performed for Bayesian inference in MrBayes v.3.2.2 (Ronquist et al. 2012) online. The Markov chain Monte Carlo (MCMC) sampling in MrBayes v.3.2.2 was used to determine the posterior probabilities (PP). Six simultaneous Markov chains were run for 1,000,000 generations, and trees were sampled every 1,000^th^ generation. The first 25% of the trees were discarded as burn-ins. The remainder was used to calculate the posterior probabilities (PPs) for individual branches. The phylogenetic tree was visualized in FIGTREE v.1.4.4 (Rambaut 2018). All analyses were run on the CIPRES Science Gateway v.3.3 web portal (Miller et al. 2010). All the obtained sequences were deposited in GenBank (Tables 1, 2).

**Table 1.** Information and GenBank accession numbers about species used for the phylogenetic tree (Fig. 1).

Species	Voucher / information	Host / Substrate	GenBank Accession Number				Reference
			ITS	LSU	*rpb2*	*tub*	
*Barrmaeliaoxyacanthae*	CBS 142770	*Salixcaprea*	MF488988	NA	MF488997	MF489016	Voglmayr et al. (2018)
*Barrmaeliarhamnicola*	CBS 142772^T^	*Rhamnusalpina*	MF488991	NA	MF489000	MF489019	Voglmayr et al. (2018)
*Vamsapriyaaquatica*	DLUCC:970^T^	bamboo	MZ420740	NA	NA	NA	Bao et al. (2021)
*Vamsapriyabambusicola*	MFLUCC 11-0477	bamboo	KM462835	KM462836	KM462834	KM462833	Dai et al. (2014)
	MFLUCC 11-0637	bamboo	KU940159	KU863147	KU940182	NA	Dai et al. (2017)
*Vamsapriyabreviconidiophora*	MFLUCC 14-0436	bamboo	MF621584	MF621588	NA	NA	Direct Submission
*Vamsapriyachiangmaiensis*	MFLUCC 21-0065	bamboo	MZ613171	MZ613168	NA	NA	Sun et al. (2021)
*Vamsapriyaclypeata*	GMB 5630^T^	bamboo	PQ884676	PQ885388	NA	PQ893585	Liu et al. (2025)
	GMB 5642	bamboo	PQ884677	PQ885389	NA	PQ893586	Liu et al. (2025
*Vamsapriyadamingshanensis*	GMB 5627^T^	bamboo	PQ884680	PQ885392	NA	PQ893589	Liu et al. (2025)
	GMB 5643	bamboo	PQ884681	PQ885393	NA	PQ893590	Liu et al. (2025)
***Vamsapriyazhaotongensis***	**GMB6401**	**bamboo**	**PV637143**	**PV637137**	**PV642220**	**NA**	**This study**
	**GMB6402**	**bamboo**	**PV6437144**	**PV637138**	**PV642221**	**NA**	**This study**
*Vamsapriyaindica*	MFLUCC 12-0544^T^	bamboo	KM462839	KM462840	KM462841	KM462838	Dai et al. (2014)
	MFLUCC 21-0088	bamboo	MZ613172	MZ613169	OK560921	NA	Sun et al. (2021)
*Vamsapriyakailiensis*	GMB6237	bamboo	PQ874043	PQ860492	NA	PQ864006	Habib et al. (2025)
	GMB6236^T^	bamboo	PQ874042	PQ860491	NA	PQ864005	Habib et al. (2025)
*Vamsapriyakhunkonensis*	MFLUCC 13-0497^T^	bamboo	KM462830	KM462831	KM462829	KM462828	Dai et al. (2014)
	MFLUCC 11-0475	bamboo	MW240620	MW240549	KM462829	KM462828	Dai et al. (2014)
*Vamsapriyamucosa*	MFLU 18-0103^T^	bamboo	MW240622	MW240551	MW658614	MW775580	Samarakoon et al (2022)
*Vamsapriyashiwandashanensis*	GMB 5632^T^	bamboo	PQ884678	PQ885390	PQ893613	PQ893587	Liu et al. (2025)
	GMB 5641	bamboo	PQ884679	PQ885391	PQ893614	PQ893588	Liu et al. (2025)
***Vamsapriyatongluobaensis***	**GMB6404**	**bamboo**	**PV637141**	**PV637135**	**PV642218**	**PV642222**	**This study**
	**GMB6405**	**bamboo**	**PV637142**	**PV637136**	**PV642219**	**PV642223**	**This study**
*Vamsapriyasichuanensis*	HKAS 130304^T^	bamboo	PP584757	PP584830	PP993507	NA	Dissanayake et al (2024)
*Vamsapriyauniseptata*	GZCC 21-0892^T^	bamboo	MZ613173	MZ613170	NA	NA	Sun et al. (2021)
	MFLU23-0261	bamboo	OR259106	OR259087	NA	OR269618	Sun et al. (2021)
*Vamsapriyayadongensis*	HKAS 134926	wood	PQ350312	PQ350314	PQ563198	PQ563196	Wang et al. (2024)
*Vamsapriyayunnana*	KUMCC 18-0008^T^	bamboo	MG833874	MG833873	MG833875	NA	Jiang et al. (2022)

Notes: Type specimens are marked with T; “NA” indicates no sequence available in GenBank; newly generated sequences are indicated in bold.

**Table 2.** Information and GenBank accession numbers of species used in phylogenetic tree (Fig. 2).

Species	Voucher / information	Host / Substrate	GenBank Accession Number			Reference
			LSU	SSU	ITS	
*Chaetosphaeriachlorotunicata*	SMH 1565^T^	decorticated wood	AF466064	NA	NA	Miller and Huhndorf (2004)
*Chloridiumlignicola*	CBS 143.54^T^	soil	MH868806	NA	MH857273	Vu et al. (2019)
*Dictyochaetafuegiana*	FMR_13126	leaves	KY853500	NA	KY853440	Spegazzini (1923)
*Echinosphaeriacanescens*	SMH 4666^T^	dead branches	KF765605	NA	NA	Miller and Huhndorf (2004)
*Endophragmielladimorphospora*	FMR_12150	soil	KY853502	HF937351	KY853442	Hernández-Restrepo et al. (2017)
*Exserticlavavasiformis*	TAMA 450	rotten wood	AB753846	NA	NA	Lin et al. (2019)
*Gelasinosporatetrasperma*	CBS 178.33^T^	soil	DQ470980	DQ471032	NA	Cai et al. (2006)
*Helminthosphaeriaclavariarum*	SMH 4609^T^	soil	AY346283	NA	NA	Huhndorf et al. (2004)
*Hilberinacaudata*	SMH 1542^T^	rotten wood	KF765615	NA	NA	Miller et al. (2014)
*Leptosporellaarengae*	MFLUCC 15-0330^T^	*Arengapinnata*	MG272246	NA	MG272255	Konta et al. (2017)
*Leptosporellabambusae*	MFLUCC 12-0846^T^	bamboo	KU863122	NA	KU940134	Dai et al. (2017)
*Leptosporellacocis*	MFLUCC 15-0816^T^	cocoies	NA	MG366595	MG272256	Dai et al. (2017)
*Leptosporellagregaria*	SMH 4290^T^	rotten wood	AY346290	NA	NA	Huhndorf et al. (2004)
	SMH 4673	rotten wood	HM171287	NA	NA	Huhndorf and Miller (2011)
*Linocarponarengae*	MFLUCC 15-0331^T^	*Arengapinnata*	MG272247	MG272252	NA	Konta et al. (2017)
*Linocarponcocois*	MFLUCC 15-0812^T^	*Cocosnucifera*	MG272248	MG272253	MG272257	Konta et al. (2017)
*Neolinocarponarengae*	MFLUCC 15-0323^T^	*Arengapinnata*	MG272249	MG366597	MG272258	Konta et al. (2017)
***Neolinocarponbambusicola***	**GMB6407**	**bamboo**	**PV637139**	**PV637147**	**PV637145**	**This study**
	**GMB6408**	**bamboo**	**PV637140**	**PV637148**	**PV637146**	**This study**
*Neolinocarponenshiense*	HKUCC2983	dead petiole	DQ810221	NA	NA	Bahl (2006)
*Neolinocarponglobosicarpum*	HKUCC2983	decaying intertidal fronds	DQ810224	DQ810258	NA	Hyde and Alias (1999)
*Neolinocarponhuaxiense*	GMB6204	*bamboo*	PQ860477	PQ860578	PQ874031	Habib et al. (2025)
	GMB6203^T^	*bamboo*	PQ860476	PQ860577	PQ874030	Habib et al. (2025)
*Neolinocarponphayaoense*	MFLUCC 17-00073a	*Heveabrasiliensis*	MG581933	MG581936	NA	Senwanna et al. (2018)
	MFLUCC 17-00073b	*Heveabrasiliensis*	MG581934	MG581937	NA	Senwanna et al. (2018)
	MFLUCC 17-00074^T^	*Heveabrasiliensis*	MG581935	MG581938	NA	Senwanna et al. (2018)
*Neolinocarponrachidis*	MFLUCC 15-0332^T^	*Arengapinnata*	MG272250	MG366598	NA	Konta et al. (2017)
	MFLUCC 15-0814a	*Cocosnucifera*	MK106353	MK106367	MK106342	Konta et al. (2017)
	MFLUCC 15-0814b	*Cocosnucifera*	MK106354	MK106368	NA	Konta et al. (2017)
*Ruzeniaspermoides*	SMH4606	rotten wood	AY436422	NA	NA	Miller and Huhndorf (2004)
*Sordariafimicola*	CBS 508.50	wood	AY681160	NA	AY681188	Niranjan and Sarma (2021)
*Synaptosporaplumbea*	SMH 3962	wood	KF765621	NA	NA	Miller et al. (2014)
*Umbrinosphaeriacaesariata*	CBS 102664	decayed wood	AF261069	NA	NA	Réblova and Winka (2001)
*Zanclosporaiberica*	FMR_11584^T^	wood	KY853544	NG_070789	KY853480	Hernández-Restrepo et al. (2017)

Notes: Type specimens are marked with T; “NA” indicates no sequence available in GenBank; newly generated sequences are indicated in bold.

## ﻿Results

### ﻿Phylogenetic analyses 1

**Figure 1.**
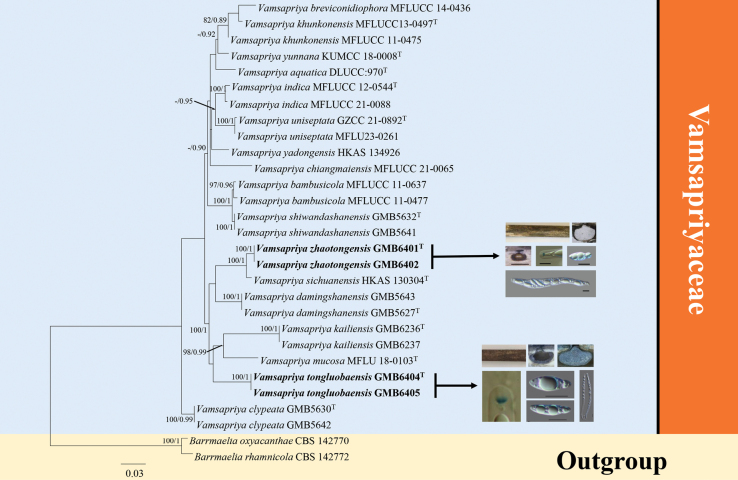
RAxML tree of *Vamsapriya* and its related taxa based on a combined LSU, ITS, *rpb2*, and *tub2* sequences dataset. Bootstrap support values for maximum likelihood (MLB) greater than 75% and Bayesian posterior probabilities (BYPP) greater than 0.90 are displayed above the respective branches (ML/BI). The newly described species are marked in bold.

The aligned dataset of Vamsapriyaceae (Fig. 1), comprising 3,080 characters (ITS/LSU/*tub2*/*rpb2*), contains 29 strains, including the newly generated sequences and outgroup taxa. *Barrmaeliaoxyacanthae* and *B.rhamnicola* were selected as outgroup taxa. The tree topology from the ML analysis was similar to that from the BI analysis. The best-scoring RAxML tree is presented in Fig. 1. The sequences of our specimen, *Vamsapriyazhaotongensis*, formed a well-supported (ML/BI: 100/1.00) clade in a sister relationship with *V.sichuanensis*. The lineage branch of this clade contains the second collection, *Vamsapriyatongluobaensis*, which shows a close relationship with *V.mucosa* and *V.kailiensis*. Although this clade lacks robust support, it remained stable in repeated phylogenetic analyses.

### ﻿Phylogenetic analyses 2

**Figure 2.**
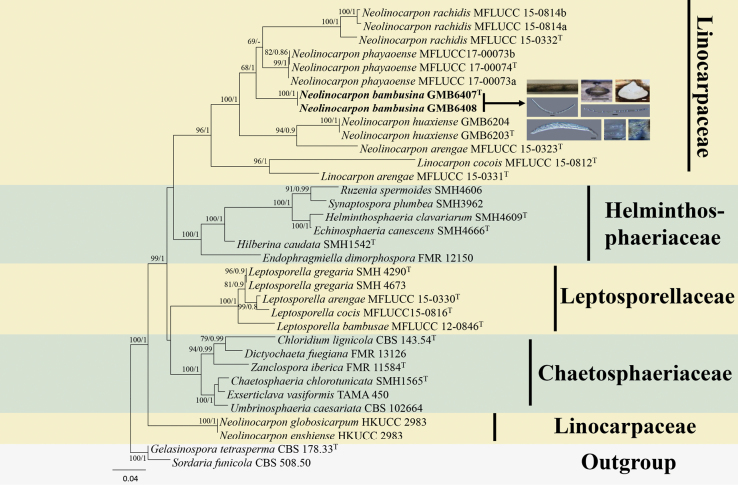
Phylogenetic tree based on combined partial SSU, LSU, and ITS sequences showing the relationship of one new species of *Neolinocarpon* from Yunnan Province with other species. Numbers at the branches indicate support values (RAxML-BS/BI-PP) above 60%/0.9. Ex-type materials are marked with “T.” Materials in bold type are those analyzed in this study.

The aligned dataset of Linocarpaceae comprised 2,454 characters (ITS/LSU/SSU) after exclusion of ambiguously aligned regions and long gaps. *Sordariafimicola* and *Gelasinosporatetrasperma* were selected as outgroup taxa. In our phylogram (Fig. 2), the sequences of our collection, *Neolinocarponbambusicola*, nested within the phylogenetic branch of the genus *Neolinocarpon*, formed a separate clade outside a group comprised of *N.phayaoense* and *N.rechedis*, demonstrating its status as an independent species.

### ﻿Taxonomy

#### ﻿ Vamsapriya zhaotongensis

Taxon classificationFungiXylarialesVamsapriyaceae

X. Y. Luo, K. Habib & Q. R. Li sp. nov.

11FFEA35-6A59-5770-B13E-46ADC72A80B8

859145

Fig. 3

##### Etymology.

The specific epithet “zhaotongensis” refers to the location, Zhaotong City, where the holotype specimen was collected.

**Figure 3.**
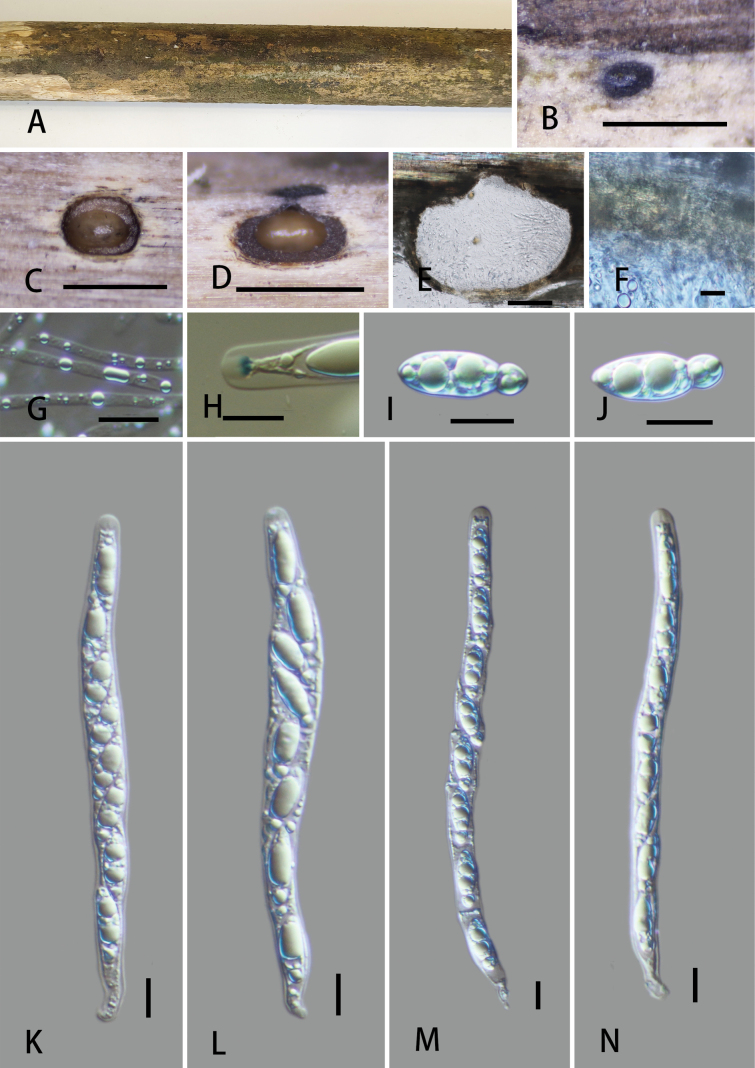
*Vamsapriyazhaotongensis* (GMB6401, holotype). A. Type material; B. Ascomata on the host surface; C. Cross-section of ascoma; D, E. Longitudinal sections of ascomata; F. Peridium; G. Paraphyses; H. A J+ subapical ring bluing in Melzer’s reagent; I, J. Ascospores; K–N. Asci. Scale bars: 1 mm (B–D); 100 μm (E); 10 μm (F–N).

##### Type.

China • Yunnan Province, Zhaotong City, Shuifu County, Tongluo Dam National Forest Park (28°26'20.40"N, 104°5'12.09"E), altitude: 1315 m, on dead culms of bamboo, Jun 2024, collected by Xing Yu Luo (holotype: GMB6401; isotype: KUN-HKAS 146990; ex-type living culture: GMBC6401).

##### Description.

Saprobic on dead bamboo culm. ***Sexual morph***: Ascomata 560–675 × 720–800 μm (x̄ = 606 × 761 μm, n = 5), immersed, visible as black, circular dots, solitary, scattered, in cross-section subglobose, with mostly flattened base. Ostioles centric, ostiolar canal periphysate. Peridium 12–17 μm (x̄ = 14.1 μm, n = 10) wide, two-layered, outer layer comprising dark brown, thick-walled cells of *textura angularis*, inner layer composed of hyaline, thin-walled cells of *textura angularis*. Paraphyses 1.7–4.2 μm (x̄ =3.3 μm, n = 30) wide, longer than asci, numerous, guttulate. Asci 125–180 × 8–14.5 μm (x̄ = 151.1 × 10.7 μm, n = 25), 8-spored, unitunicate, cylindrical, short pedicellate, with a 1.6–2.7 × 3.0–4.2 μm (x̄ = 2.1 × 3.4 μm, n = 30), trapezoid apical ring, J+ in Melzer’s reagent, apex rounded. Ascospores 17.5–21 × 5.4–8.2 μm (x̄ = 19.5 × 6.7 μm, n = 30), uniseriate, hyaline, ellipsoidal to broadly fusiform, rounded at both ends, apiosporous; smaller cell 4.4–5.1 μm (x̄ = 4.7 μm, n = 30) long, larger cell 13–16 μm (x̄ = 14.8 μm, n = 30) length, usually with large guttules, lacking mucilaginous sheath. ***Asexual morph***: Undetermined.

##### Culture characters.

Ascospores cultured on PDA medium at 27 °C for 4–5 weeks, colony diameter 4–4.5 cm, circular, cottony, slightly raised in center, with a distinct margin. White from above; pale brown from below, white in center.

##### Paratype.

China • Yunnan Province, Zhaotong City, Shuifu County, Tongluo Dam National Forest Park (28°26'14.07"N, 104°5'20.06"E), altitude: 1253 m, on bamboo, Jun 2024, collected by Xingyu Luo (GMB6402; GMBC6402).

##### Notes.

Phylogenetically, *Vamsapriyazhaotongensis* is closely related to *V.sichuanensis*. In the BLAST search, the closest match for the ITS sequence of *Vamsapriyazhaotongensis* was *V.sichuanensis* (HKAS 130304) with 95.59% similarity, followed by *V.damingshanensis* (GMB5627) with 89.84% similarity. The LSU sequences of *V.zhaotongensis* showed 99.51% similarity to *V.sichuanensis* (HKAS 130304) and 98.39% to *V.damingshanensis* (GMB5627). The *rpb2* sequences showed 99.59% similarity to *V.sichuanensis* (HKAS 130304). Morphologically, *V.sichuanensis* can be easily distinguished from *V.zhaotongensis* in having ascospores surrounded by a mucilaginous sheath. Moreover, the *V.sichuanensis* has smaller stromata (250–400 × 350–500 μm vs. 560–675 × 720–800 μm) and smaller asci (100–160 × 7–10 μm vs. 125.9–180.9 × 8.0–14.3 μm). *Vamsapriyadamingshanensis* differs from *V.zhaotongensis* in having larger ascospores (21–25 × 7–12.5 µm vs. 17.5–21 × 5.4–8.2 μm), with a large cell (16.4–19 μm long vs. 13–16 μm long) and basal cell (5–8.5 μm long vs. 4.4–5.1 μm long), and ascospores of *V.damingshanensis* surrounded by a thin gelatinous mucilaginous sheath vs. lacking in *V.zhaotongensis*.

#### ﻿ Vamsapriya tongluobaensis

Taxon classificationFungiXylarialesVamsapriyaceae

X.Y. Luo, K. Habib & Q.R. Li sp. nov.

A8A590BA-A13B-55C4-8BC3-928BDE2C7539

859144

Fig. 4

##### Etymology.

The specific epithet “tongluobaensis” refers to the geographical location, Tongluo Dam National Forest Park, where the holotype specimen was collected.

**Figure 4.**
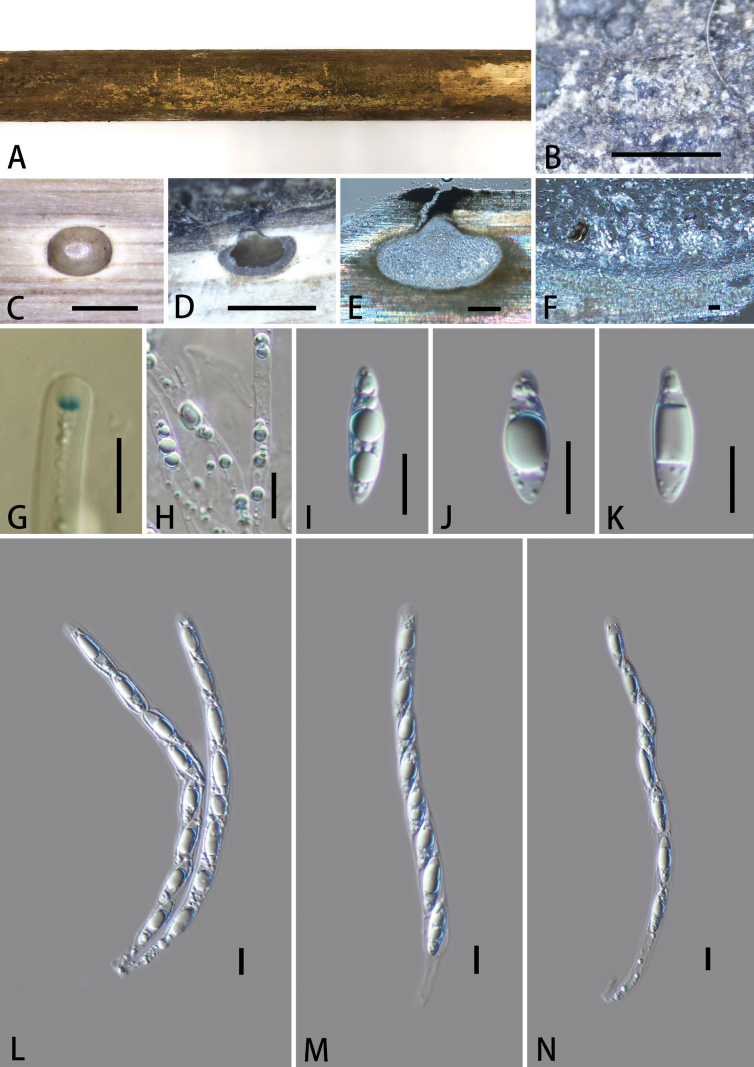
*Vamsapriyatongluobaensis* (GMB6404, holotype). A. Type material; B. Ascomata on the host surface; C. Cross-section of ascoma; D, E. Longitudinal sections of ascomata; F. Peridium; G. A J+ subapical ring bluing in Melzer’s reagent; H. Paraphyses; I–K. Ascospores; L–N. Asci. Scale bars: 0.5 mm (B–D); 100 μm (E); 10 μm (F–N).

##### Type.

China • Yunnan Province, Zhaotong City, Shuifu County, Tongluo Dam National Forest Park (28°26'18.26"N, 104°5'20.28"E), altitude: 1306 m, on dead culms of bamboo, Jun 2024, collected by Xingyu Luo (holotype: GMB6404; isotype: KUN-HKAS 146991; ex-type living culture: GMBC6404).

##### Description.

Saprobic on dead bamboo culms. ***Sexual morph***: Ascomata 600–875 × 440–610 μm (x̄ = 791 × 550 μm, n = 5), immersed, visible as black dots, with clypeus-solitary, scattered, in cross-section globose to subglobose. Ostioles centric, raised, ostiolar canal periphysate. Peridium 46–133 μm (x̄ = 83.7 μm, n = 10) wide, two-layered, outer layer comprising dark brown, thick-walled cells of *textura angularis*, inner layer composed of hyaline, thin-walled cells of *textura angularis*. Paraphyses 2.8–6.2 μm (x̄ = 4.3 μm, n = 30) wide, long, septate, constricted at septa, guttulate. Asci 115.5–170 × 7.6–11.5 μm (x̄ = 146.5 × 8.9 μm, n = 25), 8-spored, unitunicate, cylindrical, short pedicellate, with a 2–3.4 × 1.5–2.3 μm (x̄ = 3.1 × 1.9 μm, n = 30), trapezoid apical ring, J+ in Melzer’s reagent. Ascospores 15–24 × 4.4–6.8 μm (x̄ = 19.3 × 5.9 μm, n = 30), L/W 3.3, uniseriate, hyaline, ellipsoidal, apiosporous; smaller cell 2.8–5.1 μm (x̄ = 3.7 μm, n = 30) long, conical; larger cell 13.4–21 μm (x̄ = 15.6 μm, n = 30) length, usually with large guttules, lacking mucilaginous sheath. ***Asexual morph***: Undetermined.

##### Culture characters.

Ascospores cultured on PDA medium at 27 °C for 3–4 weeks, colony diameter 4–5 cm, circular, cottony surface, slightly raised in center, with a distinct margin. White from above; yellow-white to pale brown from below.

##### Paratype.

China • Yunnan Province, Zhaotong City, Shuifu County, Tongluo Dam National Forest Park (28°26'22.16"N, 104°5'10.06"E), altitude: 1411 m, on dead bamboo culms, Jun 2024, collected by Xingyu Luo (GMB6405; paratype living culture: GMBC6405).

##### Note.

Phylogenetically, *Vamsapriyatongluobaensis* is closely related to *V.mucosa*. In the BLAST search, the closest match of the ITS sequences of *V.tongluobaensis* showed 89.37% similarity to *V.mucosa* MFLU 18-0103, followed by *V.kailiensis* GMB6236 with 85.90% similarity. Its LSU sequences demonstrated 98.23% and 97.66% similarity to *V.mucosa* (MFLU 18-0103) and *V.kailiensis* (GMB6236), respectively, while its *rpb2* sequences showed 91.64% similarity to *V.mucosa* (MFLU 18-0103). Morphologically, *V.mucosa* differs in having smaller ascomata (260–300 × 320–380 μm vs. 600–875 × 440–610 μm), smaller asci (80–95 × 5–7.5 μm vs. 115.5–170 × 7.6–11.5 of *V.tongluobaensis*), smaller ascospores (10–14 × 3.5–4.5 μm vs. 15–24 × 4.4–6.8 μm of *V.tongluobaensis*), and ascospores surrounded by a 4–10 μm wide mucilaginous sheath vs. lacking in *V.tongluobaensis* (Samarakoon et al. 2022).

Morphologically, *Vamsapriyazhaotongensis* resembles *V.tongluobaensis* in the lack of a mucilaginous sheath around the ascospores. However, *V.zhaotongensis* differs in having shorter ascospores (17.5–21 × 5.4–8.2 μm vs. 15–24 × 4.4–6.8 μm of *V.tongluobaensis*) with a ratio of basal to apical cell in *V.zhaotongensis* of 3.1, while in *V.tongluobaensis*, it is 4.2. Additionally, the ITS sequences of *Vamsapriyatongluobaensis* show 90.94% similarity to *V.zhaotongensis*. LSU sequences show 98.17% similarity. Based on these morphological differences and phylogeny, we introduce *V.tongluobaensis* as a new species.

#### ﻿ Neolinocarpon bambusicola

Taxon classificationFungiChaetosphaerialesLinocarpaceae

X.Y. Luo, K. Habib & Q.R. Li sp. nov.

2E5BC92E-513D-5135-A9F5-9EE14BC59945

859146

Fig. 5

##### Etymology.

The specific epithet “bambusicola” refers to the host genus *Bambusa* (Poaceae) on which the holotype specimen was collected.

**Figure 5.**
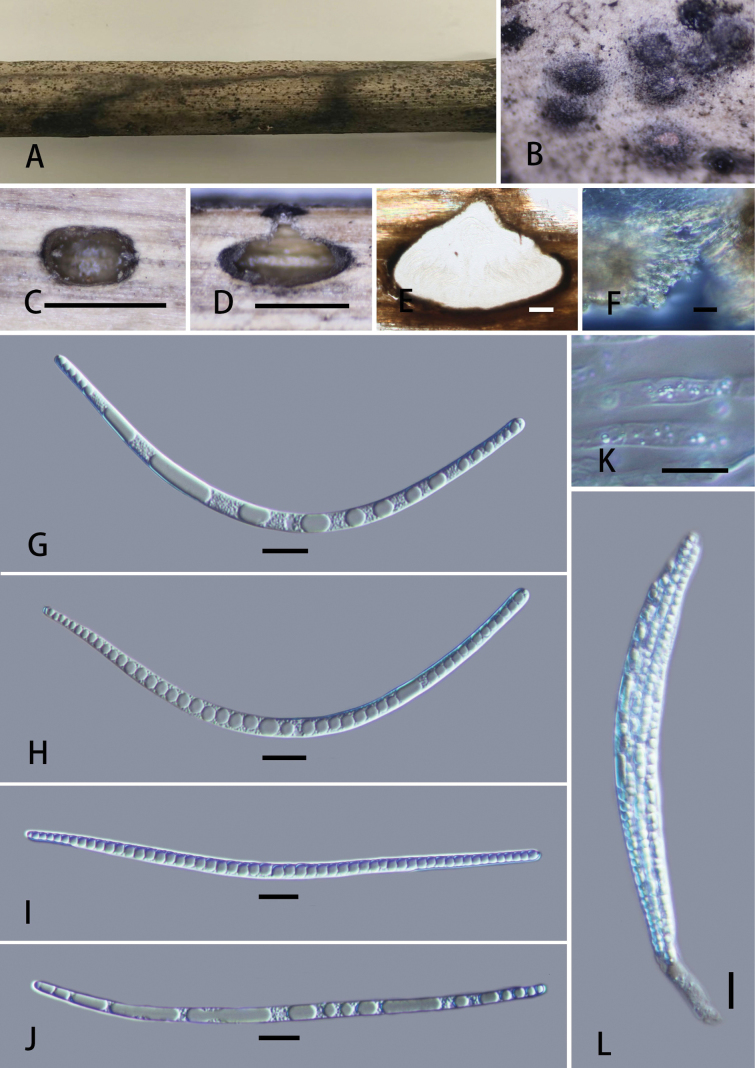
*Neolinocarponbambusicola* (GMB6407; holotype) A. Type material; B. Ostioles; C. Cross-section of ascoma; D, E. Longitudinal sections of ascomata; F. Peridium; G–J. Ascospores; K. Paraphyses; L. Asci. Scale bars: 1 mm (C, D); 100 μm (E); 10 μm (F–L).

##### Type.

China • Yunnan Province, Zhaotong City, Shuifu County, Tongluo Dam National Forest Park (28°26'14.48"N, 104°5'22.40"E), altitude: 1273 m, on dead culms of bamboo, Jun 2024, collected by Xingyu Luo (holotype: GMB6407; isotype: KUN-HKAS 146992; ex-type living culture: GMBC6407).

##### Description.

Saprobic on dead bamboo culm. ***Sexual morph***: Ascomata 410–690 × 380–745 µm (x̄ = 519 × 576 µm, n = 5), immersed, perithecial, solitary, superficial, globose to subglobose, black, clypeus slightly raised. ***Ostioles*** papillate, central, black. Peridium 19–55 μm wide (x̄ = 40 μm, n = 10), outer cells merging with host epidermal cells, composed of dark brown to black cells of *textura angularis*. Paraphyses 2.5–4 µm wide (x̄ = 3 μm, n = 20), numerous, hyaline, hypha-like, long-cylindrical, unbranched, septate, longer than asci. Asci 142–230 × 10–15 μm (x̄ = 176.8 × 13.3 μm, n = 25), 8-spored, unitunicate, cylindrical, short-pedicellate, with a J-, wedge-shaped, subapical ring. Ascospores 106–144 × 3–5 μm (x̄ = 130 × 4.5 μm, n = 30), filiform, straight or curved, hyaline, aseptate, containing numerous refringent septum-like bands up to 50, ends rounded, lacking polar mucilaginous appendages, smooth-walled. ***Asexual morph***: Undetermined.

##### Culture characteristics.

Ascospores germinating on PDA within 24 h. Colonies on PDA reached 50 mm in diameter after one month at 27 °C, colonies circular, edge entire, velvety to woolly, from above white, below yellow-white to light brown.

##### Paratype.

China • Yunnan Province, Zhaotong City, Shuifu County, Tongluo Dam National Forest Park (28°26'20.44"N, 104°5'16.28"E), altitude: 1098 m, on bamboo, Jun 2024, collected by Xingyu Luo (GMB6408; paratype living culture: GMBC6408).

##### Note.

Phylogenetic analysis (Fig. 2) based on ITS, LSU, and SSU sequences revealed *Neolinocarponbambusicola* to be closely related to *N.phayaoense*. In the BLAST search, the closest match for the LSU sequence of *N.bambusicola* was *N.phayaoense* (MFLUCC 17-00073a) with 93.2% similarity, followed by *N.arengae* (MFLUCC 15-0323) with 91.5% similarity. The SSU sequence of *N.bambusicola* showed 98.88% similarity to *N.phayaoense* (MFLUCC 17-00073a) and 99.29% similarity to *N.arengae* (MFLUCC 15-0323).

Morphologically, *Neolinocarponbambusicola* resembles *N.phayaoense* in having filiform ascospores lacking mucilaginous appendages. However, *N.phayaoense* can be distinguished from *N.bambusicola* by its host *Heveabrasiliensis* in Euphorbiaceae vs. bamboo, smaller ascomata with 1–3 locules (250–550 × 120–300 μm vs. 410–690 × 380–745 µm), longer asci (115–130 μm vs. 142–230 μm), and smaller ascospores (77–92 × 2–5 μm, x̄ = 85 × 4 μm vs. 106–144 × 3–5 μm, x̄ = 130 × 4.5 μm) (Senwanna et al. 2018).

Morphologically, *Neolinocarponbambusicola* resembles *N.arengae* in ascal size and the lack of mucilaginous appendages on ascospores. However, the latter differs in host preference, being reported on dead leaflets of *Arengapinnata* (a palm, Arecaceae), and in having slightly smaller ascospores (114–134 × 3–4 μm), whereas *N.bambusicola* is found on dead bamboo culms (Poaceae) and has slightly larger ascospores (106–144 × 3–5 μm). Although both species occur on monocotyledonous hosts, the two species are phylogenetically distinct. Based on these morphological differences and phylogenetic evidence, we introduce *N.bambusicola* as a new species (Konta et al. 2017).

## ﻿Discussion

China possesses the richest bamboo resources in the world, accounting for about one-third of the global bamboo forest area (Dlamini et al. 2022). This ecological dominance is reflected in its rich fungal diversity and hosts over one-third of the world’s documented bambusicolous Ascomycota (Jiang et al. 2022). Up to 2022, 174 bambusicolous ascomycete taxa had been documented in Southwest China, representing approximately 34% of China’s total known bambusicolous Ascomycota (Jiang et al. 2022). Since then, about 150 new bambusicolous ascomycete species have been described from China, mostly from southwestern regions (Liang et al. 2023; Wu et al. 2023; Yu et al. 2023; Dissanayake et al. 2024; Habib et al. 2024; Li et al. 2024a, b; Shen et al. 2024; Yu et al. 2024a, b; Zhou et al. 2024; Liu et al. 2025). This remarkable increase highlights the hyperdiversity of bambusicolous fungi in China, and Yang et al. (2024) reported that bamboo forests in southwestern China are still unexplored for bamboo-associated fungi, with many species yet to be discovered.

This study contributes to the knowledge of bambusicolous fungal diversity in southern China through the description of three novel species assigned to the genera *Vamsapriya* and *Neolinocarpon*. All species of *Vamsapriya* have been reported on bamboo, except *V.yadongensis*, whose host remains unidentified. The two new species described in this study were also found on bamboo, suggesting that the genus may exhibit a strong host specificity. Globally, 14 species of *Vamsapriya* have been documented, and among these, eight species are known to possess a sexual morph (Dai et al. 2017; Sun et al. 2021; Samarakoon et al. 2022; Dissanayake et al. 2024; Liu et al. 2025). In this study, we added another *Vamsapriya* species from its sexual morph. The species in this genus with sexual morphs can be divided into two sections: those with a mucilaginous sheath on the ascospore and those without. Among the previously known species, *V.clypeata*, *V.kailiensis*, and *V.shiwandashanensis* lack a mucilaginous sheath, whereas *V.bambusicola*, *V.chiangmaiensis*, *V.damingshanensis*, *V.mucosa*, and *V.sichuanensis* are characterized by its presence. The species of the genus can be further differentiated by the size of ascomata and asci and the shape and size of their ascospores. The two newly recognized species of *Vamsapriya* do not possess a mucilaginous sheath. Among the species lacking a sheath on the ascospores, *V.kailiensis* possesses the smallest ascospores in the genus (<14 µm), whereas *V.shiwandashanensis* has the largest (20–29 × 5.5–7.5 µm). *Vamsapriyaclypeata* can be confused with *V.zhaotongensis* because of their similar ascospore size (18.5–21 × 7.2–8.9 µm) and shape. However, *V.zhaotongensis* differs in having slightly smaller asci (115.5–170 × 7.6–11.5 µm vs. 129–181 × 8.2–9.6 µm in *V.clypeata*) and is phylogenetically distantly related. *Vamsapriyatongluobaensis* can be easily distinguished from *V.clypeata* by its larger ascospores (15–24 × 4.4–6.8 µm) pointed at both ends, vs. rounded ends in *V.clypeata* (Liu et al. 2025).

Nearly all previously known species of *Neolinocarpon* have been found on hosts in the Arecaceae, indicating a strong host preference within this genus. The exceptions are *N.penniseti*, which occurs on the dead stems of *Pennisetumpurpureum* (Poaceae), and *N.phayaoense*, found on branches of *Heveabrasiliensis* (Euphorbiaceae) (Hyde 1992b; Hyde et al. 1998; Hyde and Alias 1999; Bhilabutra et al. 2006; Vitória et al. 2013; Senwanna et al. 2018). Within the Arecaceae, *Neolinocarpon* species are reported on different plants and are associated with various plant parts. *Neolinocarponaustraliense* is associated with dead rattan of *Calamusmoti*, while *N.attaleae* occurs on dead rachises of *Attaleafunifera*. Similarly, *N.calami* and *N.enshiense* inhabit dead petioles of *Calamusconirostris* and *Trachycarpusfortunei*, respectively. *Neolinocarponglobosicarpum* and *N.nypicola* are reported on decaying intertidal fronds and intertidal petioles of *Nypafruticans*. *Neolinocarponinconspicuum* and *N.nonappendiculatum* both occur on *Archontophoenixalexandrae* but occupy different tissues: dead rachises and dead petioles, respectively (Hyde 1992b; Hyde et al. 1998; Hyde and Alias 1999; Vitória et al. 2013). Recently, a new species, *N.huaxiense*, has been reported from China, isolated from bamboo (Habib et al. 2025). This study expands the known ecological range of the genus by reporting *N.bambusicola* on dead bamboo (Poaceae) culms, marking the third documented occurrence of *Neolinocarpon* on Poaceae.

The genus *Neolinocarpon* and its type species, *Neolinocarponglobosicarpum*, were introduced by the renowned mycologist K.D. Hyde, who also established several other species within the genus, including *Neolinocarponarengae* S. Konta & K.D. Hyde, *Neolinocarponphayaoense* Senwanna & K.D. Hyde, and *Neolinocarponrachidis* S. Konta & K.D. Hyde. The holotype sequences of these latter species have been verified and form a well-supported monophyletic clade, within which our newly described species also clusters. However, phylogenetic analyses reveal inconsistencies within the genus, particularly concerning *N.globosicarpum* and *N.enshiense*, whose available sequences, derived from non-type specimens, cluster distantly from the main *Neolinocarpon* clade and do not group within the familial boundaries, rendering the genus polyphyletic. This phylogenetic incongruence highlights a systematic issue within *Neolinocarpon* that can only be resolved by sequencing the holotype of *N.globosicarpum*. A detailed molecular study, including the re-examination and sequencing of existing type specimens, is urgently needed to clarify the true phylogenetic placement of these taxa. Until this is addressed, the delimitation of *Neolinocarpon* remains provisional, and the phylogenetic positions of *N.globosicarpum* and *N.enshiense* should be interpreted with caution rather than being excluded from the genus.

These findings not only highlight the vast, yet-to-be-explored fungal diversity in China’s bamboo ecosystems but also pave the way for further research to uncover additional species and clarify their ecological roles.

## Supplementary Material

XML Treatment for Vamsapriya zhaotongensis

XML Treatment for Vamsapriya tongluobaensis

XML Treatment for Neolinocarpon bambusicola

## ﻿Additional information

### Use of AI

No use of AI was reported.

### Author ORCIDs

Kamran Habib https://orcid.org/0000-0003-2572-0306

Yulin Ren https://orcid.org/0009-0003-9063-425X

Xiangchun Shen https://orcid.org/0000-0002-4333-9106

Abdallah M. Elgorban https://orcid.org/0000-0003-3664-7853

Nalin N. Wijayawardene https://orcid.org/0000-0003-0522-5498

Qirui Li https://orcid.org/0000-0001-8735-2890

### Data availability

All of the data that support the findings of this study are available in the main text.
